# Potential application of a newly isolated phage BUCT609 infecting *Stenotrophomonas maltophilia*

**DOI:** 10.3389/fmicb.2022.1001237

**Published:** 2022-11-21

**Authors:** Ke Han, Yuqi Dong, Xiaoping An, Lihua Song, Mengzhe Li, Huahao Fan, Yigang Tong

**Affiliations:** ^1^College of Life Science and Technology, Beijing University of Chemical Technology, Beijing, China; ^2^Beijing Advanced Innovation Center for Soft Matter Science and Engineering, Beijing University of Chemical Technology, Beijing, China

**Keywords:** *Stenotrophomonas maltophilia*, bacteriophage, *podoviridae*, genomic analysis, phage thearpy

## Abstract

*Stenotrophomonas maltophilia* (*S. maltophilia*) is widely distributed in nature and frequently causes nosocomial infections. In this work, the biological characteristics and genome of a new *S. maltophilia* phage BUCT609 isolated from hospital sewage with *S. maltophilia* strain No. 3015 as host was analyzed and its therapeutic effect *in vivo* was explored. It was observed by TEM that phage BUCT609 belongs to the *Podoviridae* with a 10 nm tail structure and a capsid with a diameter of about 50 nm. It has a short latent period (about 10 min) and its burst size is 382 PFU /cell when multiplicity of infection (MOI) is 0.01. Furthermore, it has a high survival rate in the environment with a pH range from 3 to 10 and temperature range from 4°C to 55°C. The complete genome of phage BUCT609 is linear double-stranded DNA of 43,145 bp in length, and the GC content is 58%. The genome sequence of phage BUCT609 shares <45% homology with other phages. No virulence genes and antibiotic resistance genes were found in bacteriophage BUCT609. *In vivo* animal experiments showed that the survival rate of mice infected with *S. maltophilia* was significantly improved after the intranasal injection of phage BUCT609. Therefore, our study supports that phage BUCT609 could be used as a promising antimicrobial candidate for treating *S. maltophilia* infections.

## Introduction

*Stenotrophomonas maltophilia* is Gram-negative bacteria that widely exists in nature and also resides in the human respiratory and intestinal tract. The isolation rate of *S. maltophilia* was only less than *Acinetobacter* and *Pseudomonas aeruginosa* among non-fermented glycogram negative bacilli (An and Berg, 2018). As a conditioned pathogen, *S. maltophilia* is a major pathogenic bacterium for iatrogenic infection that can cause many diseases such as infections of the respiratory tract, urinary tract, and wounds. In recent years, with the extensive use of broad-spectrum antibiotics, *S. maltophilia* has become one of the most important pathogens of nosocomial infection, which brings great difficulties to clinical treatment (Gokhan Gozel et al., 2015). Phages are specific for infection and lysis of host bacteria in the environment and organisms. During the recent years, many countries have made exciting achievements in phage therapy (Bao et al., 2020) which demonstrate the unique advantages of phage therapy and greatly promote further research of phage therapy, indicating that phage therapy has a very bright application prospect (Moelling et al., 2018).

In this study, phage BUCT609 was isolated from hospital sewage. The biological and genomic characteristics of phage BUCT609 were analyzed to evaluate the infectivity and animal experiments were conducted to explore its therapeutic effect on *S. maltophilia in vivo*. This study will increase the diversity of *S. maltophilia* phage and provide a potential candidate for phage-based therapy.

## Materials and methods

### Bacterial strains and culture conditions

*S. maltophilia* strain No. 3015 in our laboratory bacteria bank was used as the host bacterium of phage BUCT609, which is a clinical strain isolated from Shanghai Public Health Clinical Center. The bacteria were cultured 5 h to reach the exponential phase in Luria-Bertani (LB) medium at 37°C.

### Isolation and purification of phage BUCT609

Phage BUCT609 was isolated from sewage in the China-Japanese Friendship Hospital of Beijing. Ten milliliters of sewage water were centrifuged at 12,000 × g for 5 min and filtered by membrane filtration with 0.22 μm pore diameter (Ding et al., 2020). The host bacteria were cultured to the exponential phase (OD_600_ = 0.7) at 37°C for phage infection. Then the filtered phage (100 μl) was mixed evenly with 500 μl of the host bacteria. The mixture was added into 5 ml LB liquid medium and incubated at 37°C for 5 h. Phage was diluted by phosphate-buffered saline (PBS) buffer and verified by the standard double-layer agar method. Eventually, a single plaque was picked up and amplified and purified three times (Chang et al., 2005).

To obtain a high titer of phage solution, 8 ml of 30% sucrose solution was slowly added to phage solution (32 ml). After centrifugation at 30,000 × g for 2 h at 4°C, the supernatant was discarded. The phage was re-suspended with 200 μl of PBS buffer.

### Transmission electron microscopy

The morphological characteristics of phage BUCT609 were observed by transmission electron microscopy (TEM). As reported previously, 10 μl of purified phages were loaded onto a copper grid for 20 min and the phage particles were negatively stained with 10 μl of 2% (w/v) phosphotungstic acid (PTA) for 10 min. The samples were dried for 5 h at room temperature. Morphology of the phage was observed by a transmission electron microscope (JEM-1200EX, Japan) at 80 kV (Li et al., 2019).

### The optimal multiplicity of infection

MOI refers to the ratio of the number of phages (PFU) to the host bacteria (CFU) and the optimal MOI is the multiplicity of infection when the phage can achieve the optimal growth state (Xing et al., 2017). Five hundred microliters of each purified phage and the host bacteria were mixed (MOI = 100, 10, 1, 0.1, and 0.01) evenly and incubated for 20 min. Then the mixture was added into fresh sterile LB liquid medium for 5 h at 37°C. After incubating, the mixture was centrifugated (12,000 × g, 5 min) and filtered to obtain the phage in the supernatant. The phage titer was determined by the standard double-layer agar method and the MOI with the highest titer is the optimal MOI.

### One-step growth curve

To evaluate the infectivity of phage BUCT609 with the latent period and the burst size, it is necessary to study the one-step growth curve of the phage (Hagens and Loessner, 2010). The latent period is defined as the stage in which the phage proliferates in the bacterial cells. The burst size refers to the ratio of the ultimate titer of phage to the number of infected bacteria in the latent period. Phage BUCT609 was mixed with *S. maltophilia* strain No. 3015 (OD_600_ = 0.7) to the optimal MOI and allowed to adsorb at 37°C for 20 min. Then the mixture was centrifugated (12,000 × *g*, 1 min). The sediment containing phage was resuspended with 20 ml of LB liquid medium. The phage titer was determined by the soft agar overlay method every 10 min during the incubation at 37°C. Three replicates were performed and the results were averaged.

### Thermal stability and pH sensitivity

To test the thermal stability of phage BUCT609, the purified phage was incubated at different temperatures (4, 37, 45, 55, 65, or 75°C) for an hour. Then, the phage titer was calculated by the method of soft agar overlay (Fan et al., 2012). Similarly, to explore the effect of pH on phage BUCT609, the phage suspensions were incubated at various pH values ranging from 1 to 12 at 37°C for an hour and the phage titer was calculated. Each experiment was performed three times and the initial phage concentration was about 10^9^ PFU/ml.

### Multilocus sequence typing and host range

Multilocus sequence typing (MLST) is a bacterial typing method based on nucleic acid sequence determination (Larsen et al., 2012). In this experiment, multiple internal fragments of housekeeping genes were amplified by PCR and sequenced to analyze the variation of strains. Using housekeeping gene primers of *S. maltophilia (atpD*, *gapA*, *guaA*, *mutM*, *nuoD*, *ppsA*, and *recA)* for PCR amplification (Supplementary Table 1) and the amplified products were sequenced and analyzed on the MLST database.

To determine phage BUCT609 host range, 13 strains of *S. maltophilia* were cultured to the exponential phase (OD_600_ = 0.7). 500 μl of each sample were added into 5 ml 0.7% LB agar medium. Then, the mixture was immediately poured into the solid medium. After solidification, 2 μl phages were added to a double-layer plate, and PBS was added on the other side as a control. The formation of plaque was observed and recorded after 9 h.

### Antimicrobial susceptibility testing

To quickly and effectively detect the sensitivity of pathogenic bacteria to various antibiotics and guide rational drug use in the clinic, the drug sensitivity test of *S. maltophilia* strain No. 3015 was carried out by disc diffusion method. The host bacteria were cultured to the exponential phase (OD_600_ = 0.7) at 37°C. 500 μl of bacteria was added into 5 ml 0.7% LB agar medium. The mixture was immediately poured into the solid medium. After solidification, the antibiotic sensitivity testing disc was placed on the plate for incubating at 37°C overnight. If the strain was sensitive to the drug, the formation of the inhibitory zone could be observed. This experiment was repeated three times.

### Phage DNA preparation

Phage genomic DNA was extracted by the Proteinase K/SDS method (Pickard, 2009). 600 μl of purified phage were incubated with DNase I and RNase A overnight at 37°C after which the enzymes were inactivated at 80°C for 15 min. 24 μl of EDTA (20 mM) with 1.5 μl of proteinase K (50 μg/ml) and 30 μl of SDS (0.5%) were added to the mixture and were incubated at 56°C for 1 h. Phage DNA was extracted with an equal volume of extraction agent (phenol: chloroform: isoamyl alcohol, 25:24:1). 400 μl of isopropanol was added to the upper aqueous layer and then the sample was incubated at −20°C for more than 1 h. 75% ethanol was added to rinse the precipitate. Eventually, the deionized water was used to dissolve the nucleic acids and supposed to be stored at −20°C.

### Whole genome sequencing and bioinformatics analysis

The whole genome was sequenced using Illumina’s MiSeq sequencing platform (Thermo Fisher Scientific, United States; Kozich et al., 2013) and the low-quality sequences were filtered by Trimmomatic (V0.32) program (Bolger et al., 2014). Then, the complete genomic sequence of BUCT609 was assembled by Newbler V3.0 software (Roche, Switzerland; Zhang et al., 2012) and CLC software (QIAGEN, Germany; Liu and Di, 2020). Using the online tools RAST (https://rast.nmpdr.org/; Aziz et al., 2008) to predict the ORFs and ORF Finder (https://www.bioinformatics.org/sms2/orf_find.html; Rombel et al., 2002) to annotate the DNA sequencing result. Sequence similarity analyses and comparisons were performed using the NCBI BLAST algorithm. A phylogenetic tree of phage BUCT609 was conducted by VICTOR (https://ggdc.dsmz.de/victor.php; Meier-Kolthoff and Göker, 2017) and average nucleotide identity (ANI) analysis was analyzed by another online tool VIRIDIC (http://rhea.icbm.uni-oldenburg.de/VIRIDIC/; Moraru et al., 2020).

### Antibacterial effect of BUCT609 *in vivo*

Specific pathogen-free (SPF) Balb/c mice reared for 6–8 weeks, weighing 17-19 g, were chosen as the animal model in this study. The median lethal dose (LD50) refers to the minimum number of bacteria required to kill half of the animal population through a specified route of infection within a specified period of time (Welkos and Brien, 1994). Pick a single clone of *S. maltophilia* No.3015 strain on the agar plate and cultivate it in LB liquid medium to exponential phase (37°C, 220 rpm). Then, the bacteria were centrifuged at 10,000 × *g* for 10 min and resuspended with PBS diluting to 8 × 10^7^ CFU/ml, 6 × 10^7^ CFU/ml, 4 × 10^7^ CFU/ml, and 2 × 10^7^ CFU/ml, respectively, for use. Forty mice were randomly divided into 5 groups (*n* = 8 in each group), of which 4 groups were used as the experimental group (injected with bacteria) and 1 group was used as the control group (injected with PBS). All mice were immunosuppressed by injection of cyclophosphamide (125 mg/kg) 4 days before infection and injected with cyclophosphamide (125 mg/kg) and dexamethasone (12.5 mg/kg) next day to stabilize the immunosuppression. After above pretreatment, each mouse was anesthetized by intraperitoneal injection of 50 mg/kg of 0.5% sodium pentobarbital. The experimental groups were injected intranasally with 40 μl of No.3015 bacterial solution with titers of 8 × 10^7^ CFU/ml, 6 × 10^7^ CFU/ml, 4 × 10^7^ CFU/ml, and 2 × 10^7^ CFU/ml, respectively, and the control group was injected with 40 μl of sterile PBS solution. The activity status of mice was observed twice a day after infection. The changes in body weight and death situation were recorded.

To explore the therapeutic effect of phage BUCT609 *in vivo*, 30 mice were equally divided into three groups (*n* = 10 in each group), two of which were the experimental group (injected with both bacteria and phage) and one was the control group (injected with PBS only). Mice were immunosuppressed and anesthetized in the same manner as described above. 40 μl of No.3015 bacterial solution was injected into the nasal cavity of the experimental group and 40 μl of PBS solution was injected into the control group. After 2 h, 40 μl of phage BUCT609 was injected into one of the experimental groups, 40 μl sterile of PBS solution was injected into other groups. The activity status of mice was observed daily after infection and the changes in body weight and death situation were recorded.

### Statistical analysis

Three replicates were performed for each experiment. Take the mean of the three sets of data and calculate the standard error. The data were subjected to one-way ANOVA test and Tukey test by GraphPad Prism 8.0.2 (GraphPad Software, Inc., La Jolla, United States). *p* < 0.05 means that the data of repeated groups are statistically significant.

## Results

### Morphology

**Figure 1:**
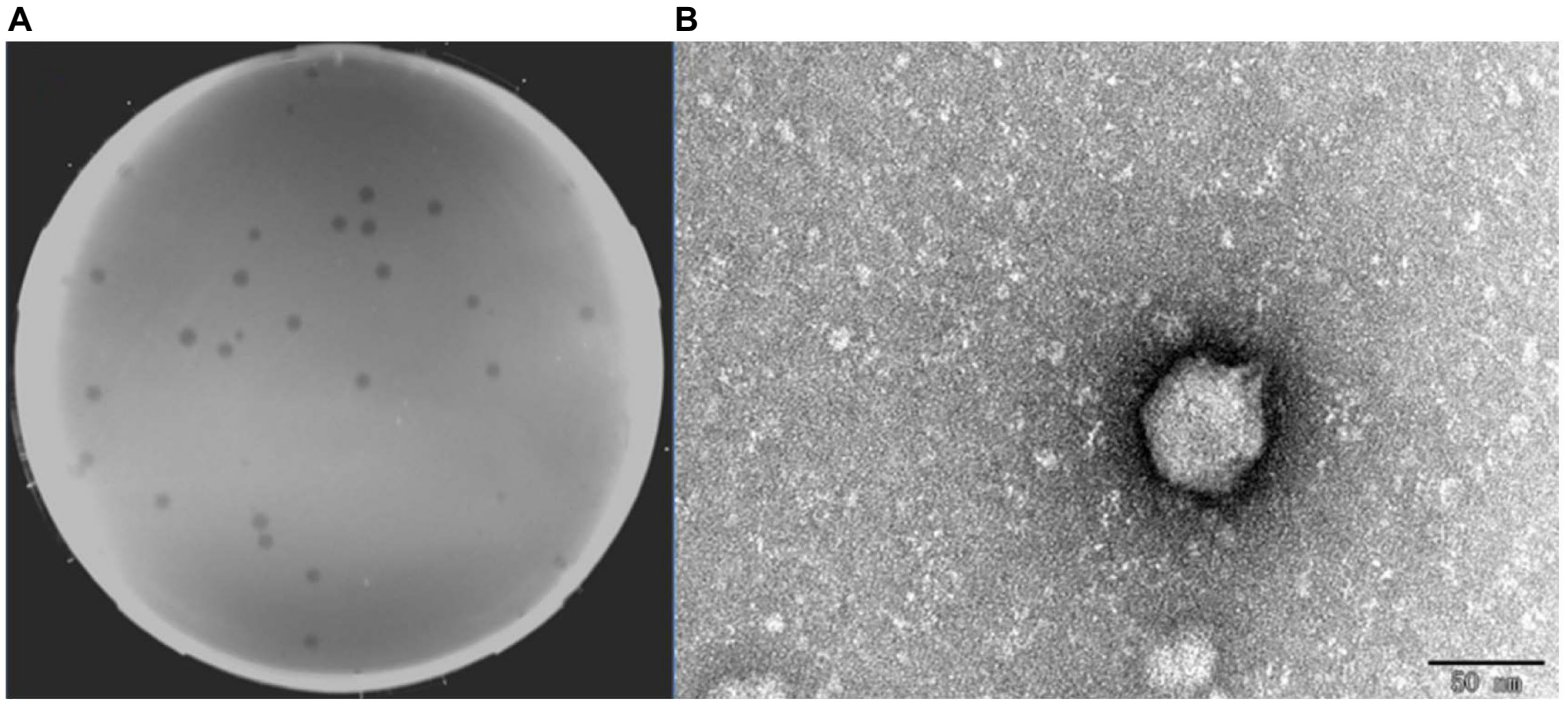
Plaques and transmission electron micrograph of phage BUCT609. **(A)** Plaques formed by BUCT609. **(B)** Transmission electron micrograph of phage BUCT609. Scale bar 50 nm.

A virulent phage BUCT609 was successfully isolated from untreated sewage in the hospital using *S. maltophilia* No. 3015 as the host bacterium. Phage BUCT609 can form transparent plaques with a diameter of 1 ~ 2 mm on a double-layer plate (Figure 1A). TEM images showed that the head diameter of phage BUCT609 was about 51.93 ± 1.68 nm, and the tail length was about 12.36 ± 1.37 nm, which can be inferred as a *Podoviridae* phage (Figure 1B).

### The optimal multiplicity of infection and One-step growth curve

Phage and host bacteria were cocultured for 5 h at different MOI ratio as shown in Supplementary Table 2 and the average titer of each phage was measured, respectively. As can be seen from Supplementary Table 2, when MOI = 0.01, the phage titer was the highest with 3.1 × 10^9^ PFU/ml. Therefore, the optimal multiplicity of infection was defined as 0.01.

**Figure 2:**
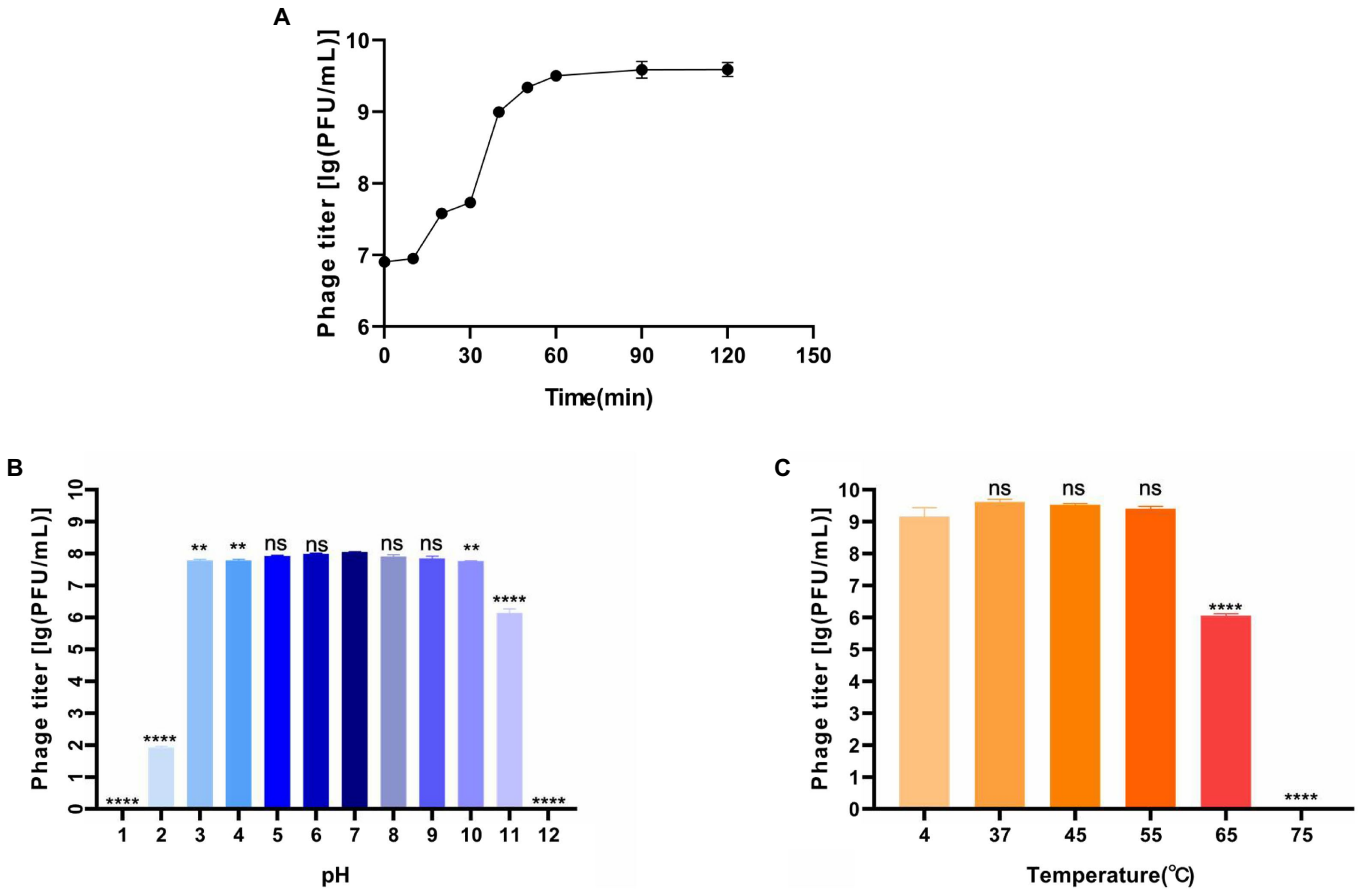
Biological characteristics of phage BUCT609. **(A)** One-step growth curve. The latent period of the phage was about 10 min, and the burst period of the phage was the following 60 min. And after 70 min, the phage grew into the plateau phase. The data is the average of three parallel experiments. Two bars above and below each point represent the standard deviation of the results of three parallel experiments. **(B)** Thermal stability. Phage titer of BUCT609 after incubation for 1 h at different temperatures. The data is the average of three replicates. There was no significant difference in the titer between 4 and 55°C. At 65 and 75°C, its titer decreased significantly (*p* < 0.0001) compared with that at 4°C. **(C)** pH sensitivity. Phage titer of BUCT609 after incubation at various pH values at 37°C for 1 h. The data is the average of three replicates. There was no significant difference in the titer from pH 5 to 9. Compared with the titer at pH 7, its titer decreased slightly (*p* < 0.01) at pH 3, 4, and 10 and decreased significantly (*p* < 0.0001) at pH 1, 2, 11, and 12. There is no significant difference between the two groups of data when *p* < 0.5, which was showed as ns in the figure. The bar above column represents the standard deviation of the results of three parallel experiments.

The one-step growth curve showed that the latent period was about 10 min (Figure 2A). In the following 60 min, the phage titer increased rapidly, where was the burst period. About 10 min later, the phage grew into the plateau phase. According to the calculation, phage BUCT609 had a large burst size (382 PFU/cell). Compared with the phage P24 (the latent period was 55 min and the burst size was 147 PFU/cell), phage BUCT609 had a shorter latent period and a larger burst size (Zhang et al., 2020). In conclusion, it was a lytic phage and had the potential to be used for phage therapy.

### Thermal stability and pH sensitivity

The thermal stability test showed that phage BUCT609 was high-temperature sensitive (Figure 2B). There was no significant difference in the titer between 4 and 55°C for 1 h. At 65°C, phage titer decreased significantly (*p* < 0.0001) compared with that at 4°C and it was completely inactivated at 75°C. As for pH tolerance (Figure 2C), phage BUCT609 exhibited a strong tolerance from pH 3 to 10. At pH 2 or 11, the phage titer was significantly lower than at pH 7 (*p* < 0.0001). And at pH 1 or 12, the phage was almost completely inactivated. Compared with phage IME392 (temperature tolerance range: 30–50°C; pH tolerance range: 4–11; Hu et al., 2021), phage BUCT609 had a wider adaptation range of temperature and pH, indicating that it had a good temperature and pH tolerance and could be broadly applied for phage therapy in the future.

### MLST and host range

The MLST results showed that 13 strains of *S. maltophilia* had different ST values, suggesting that they were 13 different species. The analysis of the host range results showed that phage BUCT609 could lyse not only *S. maltophilia* strain No. 3015 but also the other 4 *S. maltophilia* strains (No. 118, No. 548, No. 992, and No. 1207; Supplementary Table 3).

### Antimicrobial susceptibility testing

The main reason *S. maltophilia* infection is difficult to treat is that the bacteria present low susceptibility to antibiotics (Gil-Gil et al., 2020). Drug susceptibility tests confirmed that the host *S. maltophilia* No. 3015 of phage BUCT609 was resistant to a variety of antibiotics but sensitive to only a few antibiotics such as ceftriaxone, tetracycline, trimethoprim, minocycline, and levofloxacin (Supplementary Table 4).

### Characterization of phage BUCT609 genome

**Figure 3:**
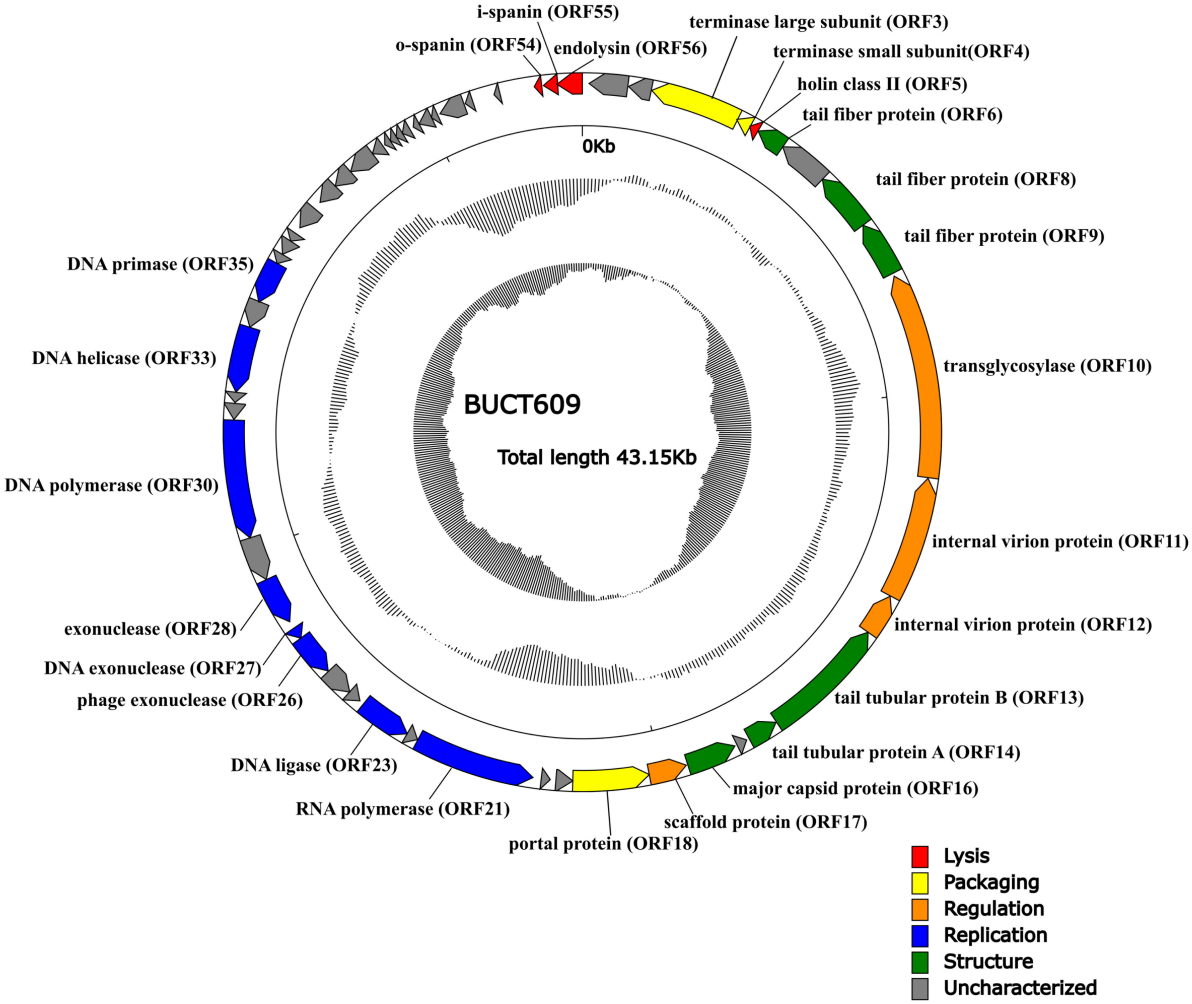
The whole genetic map of phage BUCT609. The outermost circle represents the open reading frame. Colors distinguish different functional genes while arrows represent ORF directions. The middle circle represents (G + C) mol% (outwards larger than the whole-genome average (G + C) mol%, inwards the opposite). The innermost circle represents the G + C tilt of G−C/G + C (outward for greater than 0 and inward for less than 0).

The complete sequence of phage BUCT609 was 43,145 bp in length and the CG content of the genome was 58% that had been submitted to the NCBI database with the accession number MW960043. The online comparison tool BlastN showed that it had 42% homology with *S. maltophilia* phage vB_SmeS_BUCT703 (GenBank: OM735688.1) at nucleotide level. The results of RAST online annotation showed that BUCT609 had 56 open reading frames (ORFs), of which 25 had known functions and the rest were annotated as hypothetical proteins. The majority of ORFs presented an ATG start codon (87.5%) while 2 of them started with TTG and 5 with GTG. There is no tRNA in the whole genome. The 25 functional proteins are shown in different colors on the whole genome map (Figure 3).

### Functional ORF analysis

Same as most dsDNA phage, phage BUCT609 has the same modular genomic structure such as DNA replication, regulation, phage packaging, structural, and host lytic proteins (Table 1). The structural proteins of phage BUCT609 are mainly distributed at the front of the genomic sequence. According to previous data analysis, ORF6, ORF8, ORF9, ORF13, and ORF14 were predicted to be tail related proteins. ORF14 encodes tail tubular protein A (TTPA) that is usually responsible for adhering the phage to host cells (Pyra et al., 2017), having 62.98% homology to the protein of *S. maltophilia* phage Ponderosa at amino acid level (the following homology values are all at amino acid level in this section). Besides, the major capsid protein was encoded by ORF16 and had 75.62% homology with that of *S. maltophilia* phage Ponderosa.

**Table 1:** Predicted ORFs in the genome of phage BUCT609.

ORFs	Start	Stop	Strand	Predicted Function	Best-match BLASTp Result	Accession number	E-values	Cover	Identity
ORF1	897	130	R	hypothetical protein	*Xylella* phage Paz	YP_008858922.1	1.00E-68	96%	53.36%
ORF2	1366	911	R	hypothetical protein	*Xanthomonas* phage Xaa_vB_phi31	QOI69551.1	1.00E-40	99%	50.65%
ORF3	3165	1375	R	terminase large subunit	*Stenotrophomonas* phage Ponderosa	QEG09767.1	0	99%	69.26%
ORF4	3448	3149	R	terminase small subunit	*Xanthomonas* phage Xaa_vB_phi31	QOI69549.1	3.00E-09	100%	39.00%
ORF5	3626	3438	R	holin class II	*Stenotrophomonas* phage Ponderosa	QEG09765.1	1.00E-16	98%	59.68%
ORF6	4206	3628	R	tail fiber protein	phage Titan-X	QGH45075.1	1.00E-36	99%	38.78%
ORF7	5216	4203	R	hypothetical protein	phage Titan-X	QGH45074.1	8.00E-58	100%	38.76%
ORF8	6424	5213	R	tail fiber protein	*Stenotrophomonas* phage Ponderosa	QEG09762.1	1.00E-134	70%	66.67%
ORF9	7540	6425	R	tail fiber protein	*Stenotrophomonas* phage Ponderosa	QEG09761.1	0	100%	80.65%
ORF10	11683	7601	R	transglycosylase	*Stenotrophomonas* phage Ponderosa	QEG09760.1	0	100%	69.15%
ORF11	14140	11696	R	internal virion protein	*Stenotrophomonas* phage Ponderosa	QEG09759.1	0	97%	49.69%
ORF12	14979	14149	R	internal virion protein	*Stenotrophomonas* phage Ponderosa	QEG09758.1	7.00E-94	99%	51.99%
ORF13	17519	14979	R	tail tubular protein B	*Stenotrophomonas* phage Ponderosa	QEG09757.1	0	100%	73.32%
ORF14	18149	17529	R	tail tubular protein A	*Stenotrophomonas* phage Ponderosa	QEG09756.1	7.00E-91	99%	62.98%
ORF15	18418	18203	R	hypothetical protein	*Xanthomonas* phage Xaa_vB_phi31	QOI69538.1	4.00E-14	100%	55.56%
ORF16	19464	18463	R	major capsid protein	*Stenotrophomonas* phage Ponderosa	QEG09754.1	2.00E-169	99%	72.62%
ORF17	20248	19493	R	scaffold protein	*Xylella* phage Paz	YP_008858906.1	2.00E-54	96%	44.94%
ORF18	21762	20245	R	portal protein	*Stenotrophomonas* phage Ponderosa	QEG09752.1	0	98%	71.20%
ORF19	22094	21759	R	hypothetical protein	*Stenotrophomonas* phage Ponderosa	QEG09751.1	4.00E-34	91%	72.55%
ORF20	22384	22205	R	hypothetical protein	*Xylella* phage Paz	YP_008858903.1	6.00E-15	100%	57.63%
ORF21	24947	22548	R	RNA polymerase	*Stenotrophomonas* phage Ponderosa	QEG09749.1	0	100%	71.62%
ORF22	25189	24953	R	hypothetical protein	not hits				
ORF23	26232	25186	R	DNA ligase	*Stenotrophomonas* phage Ponderosa	QEG09747.1	1.00E-75	99%	42.42%
ORF24	26592	26323	R	hypothetical protein	*Stenotrophomonas* phage Ponderosa	QEG09745.1	4.00E-15	96%	50.00%
ORF25	27173	26589	R	hypothetical protein	*Xanthomonas* phage Xaa_vB_phi31	QOI69527.1	1.00E-46	100%	41.59%
ORF26	28025	27183	R	Phage exonuclease	*Xylella* phage Cota	CAB1282933.1	6.00E-83	98%	44.40%
ORF27	28264	28022	R	DNA exonuclease	*Xanthomonas* phage XAJ24	YP_009785928.1	2.00E-27	100%	65.00%
ORF28	29363	28413	R	exonuclease	*Xanthomonas* phage Xaa_vB_phi31	QOI69524.1	4.00E-139	95%	65.12%
ORF29	30246	29365	R	hypothetical protein	*Stenotrophomonas* phage Ponderosa	QEG09740.1	2.00E-88	100%	51.01%
ORF30	32597	30243	R	DNA polymerase	*Stenotrophomonas* phage Ponderosa	QEG09739.1	0	99%	69.95%
ORF31	32926	32606	R	hypothetical protein	not hits				
ORF32	33146	32943	R	hypothetical protein	*Xylella* phage Prado	YP_008859401.1	6.00E-23	92%	69.35%
ORF33	34456	33149	R	DNA helicase	*Stenotrophomonas* phage Ponderosa	QEG09736.1	0	100%	63.53%
ORF34	34996	34457	R	hypothetical protein	not hits				
ORF35	35826	34981	R	DNA primase	*Xanthomonas* phage Xaa_vB_phi31	QOI69516.1	1.00E-131	99%	63.44%
ORF36	36032	35823	R	hypothetical protein	*Xanthomonas* phage Xaa_vB_phi31	QOI69515.1	5.00E-14	100%	66.67%
ORF37	36339	36034	R	hypothetical protein	*Xanthomonas* phage Xaa_vB_phi31	QOI69514.1	8.00E-08	89%	32.22%
ORF38	36521	36339	R	hypothetical protein	not hits				
ORF39	37135	36659	R	hypothetical protein	*Xylella* phage Paz	YP_008858885.1	3.00E-47	100%	57.86%
ORF40	37736	37296	R	hypothetical protein	*Stenotrophomonas* phage Ponderosa	QEG09730.1	9.00E-15	97%	34.23%
ORF41	38149	37736	R	hypothetical protein	not hits				
ORF42	38710	38153	R	hypothetical protein	*Stenotrophomonas* phage Ponderosa	QEG09728.1	4.00E-07	98%	35.64%
ORF43	38964	38707	R	hypothetical protein	*Stenotrophomonas* phage Ponderosa	QEG09727.1	1.00E-04	96%	33.72%
ORF44	39141	39007	R	hypothetical protein	not hits				
ORF45	39284	39144	R	hypothetical protein	not hits				
ORF46	39423	39277	R	hypothetical protein	not hits				
ORF47	39590	39420	R	hypothetical protein	*Stenotrophomonas* phage Ponderosa	QEG09722.1	5.00E-12	87%	66.04%
ORF48	39799	39668	R	hypothetical protein	not hits				
ORF49	40074	39796	R	hypothetical protein	not hits				
ORF50	40205	40071	R	hypothetical protein	not hits				
ORF51	40767	40255	R	hypothetical protein	*Stenotrophomonas* phage Ponderosa	QEG09719.1	2.00E-68	92%	68.15%
ORF52	40947	40792	R	hypothetical protein	*Xanthomonas* phage Suba	CAA2409834.1	0.011	96%	35.19%
ORF53	41505	41386	R	hypothetical protein	not hits				
ORF54	42342	42193	R	o-spanin	*Stenotrophomonas* phage Ponderosa	QEG09771.1	2.00E-13	100%	57.14%
ORF55	42658	42374	R	i-spanin	*Xylella* phage Paz	YP_008858924.1	7.00E-06	100%	30.85%
ORF56	43145	42642	R	endolysin	*Stenotrophomonas* phage Ponderosa	QEG09769.1	4.00E-66	99%	65.06%

Terminase is a key protein in phage DNA packaging that includes large subunit and small subunit. Generally, terminase large subunit and terminase small subunit are adjacent and both involved in the splicing and packaging process of phage DNA. The large subunit is responsible for ATP-driven DNA translocations while the small subunit interacts with terminase large subunit and initiate packaging through binding and cleaving specifically near the initial package site (Weiditch et al., 2019 Gao et al., 2020). Terminase small subunit specifically recognizes viral DNA, while the terminase large subunit plays an important role in ATP recognition and hydrolysis (Loredo-Varela et al., 2013). As is shown from the chart, ORF3 from phage BUCT609 encodes the terminase large subunit that has 69.26% homology with that of *S. maltophilia* phage Ponderosa. The terminase small subunit is the product of ORF14 and exhibited 39% identity to that of *Xanthomonas* phage Xaa_vb_phi31. The portal control protein is encoded by ORF18 and forms a channel at the phage tail attachment site through where the phage can inject its genome into the host cell (Prevelige Jr and Cortines, 2018).

Replication is a complex process involving a variety of proteins and enzymes. In the initiation phase, the DNA helicase encoded by OFR33 can unlock double-stranded DNA by hydrolyzing ATP for energy, with 63.53% homology to that of *S. maltophilia* phage Ponderosa. DNA primer enzyme encoded by ORF35 has 63.44% homology with that of *Xanthomonas* phage Xaa_vb_phi31, which can catalyze the synthesis of RNA primer. Different DNA polymerases play different roles in DNA replication (Hoitsma et al., 2020). Exhibiting 69.95% identity to DNA polymerase of *S. maltophilia* phage Ponderosa, the DNA polymerase encoded by ORF30 is mainly used for DNA replication and repair. Furthermore, exonuclease is a class of enzymes that degrade nucleotides one by one from the end of a polynucleotide chain (Dean et al., 2001). In phage BUCT609, ORF26, ORF27, and ORF28 encode three different exonuclease enzymes, with 44.4% homology to that of *Xylella* phage Cota, 65% homology to that of *Xanthomonas* phage Xaj24 and 65.12% homology to that of *Xanthomonas* phage XAA VB phi31, respectively. The DNA ligase encoded by ORF23 plays an important role in the process of DNA replication and repair, catalyzing the reaction that two adjacent bases connect consuming ATP and has 42.42% homology to the protein of *S. maltophilia* phage Ponderosa by comparison (Shi et al., 2018). ORF21 encodes RNA polymerase whose main function is to synthesize RNA using DNA or RNA as templates and triphosphate ribonucleoside as substrates. Due to that RNA is involved in the transcription of genetic information about genes within a cell, it is also called a transcriptase (Griesenbeck et al., 2017). The RNA polymerase ORF21 has 100% coverage but only 72% identity with that of *S. maltophilia* phage Ponderosa.

For the lytic mechanism, the phage lyses the host bacteria under the combined action of holin, lyase, and spanin. Holin encoded by OFR5 plays a crucial role in the penetration of phages after attaching to the host. Holin forms pores in the cell membrane and begins the process of osmosis and dissolution (Palmer et al., 2021). By the BlastN comparison, the holin of phage BUCT609 has 59.68% homology with *S. maltophilia* phage Ponderosa. Most phages produce a two-component protein complex consisting of outer membrane lipoprotein (o-spanin) and inner membrane protein (i-spanin) which are necessary for the host outer membrane destruction (Cahill and Young, 2019). Phage BUCT609 has an o-spanin ORF54 with 57.14% homology to the protein of *S. maltophilia* phage Ponderosa and i-spanin ORF55 with 30.85% homology to the protein of *Xylella* phage Paz. The endolysin transcribed by ORF56, which showed 65.06% identity to that of *S. maltophilia* phage Ponderosa, was synthesized by host bacteria and can result in bacteria death through inducing the lysis of bacteria cell walls specifically and effectively (Ghose and Euler, 2020).

### Phylogenetic analysis

**Figure 4:**
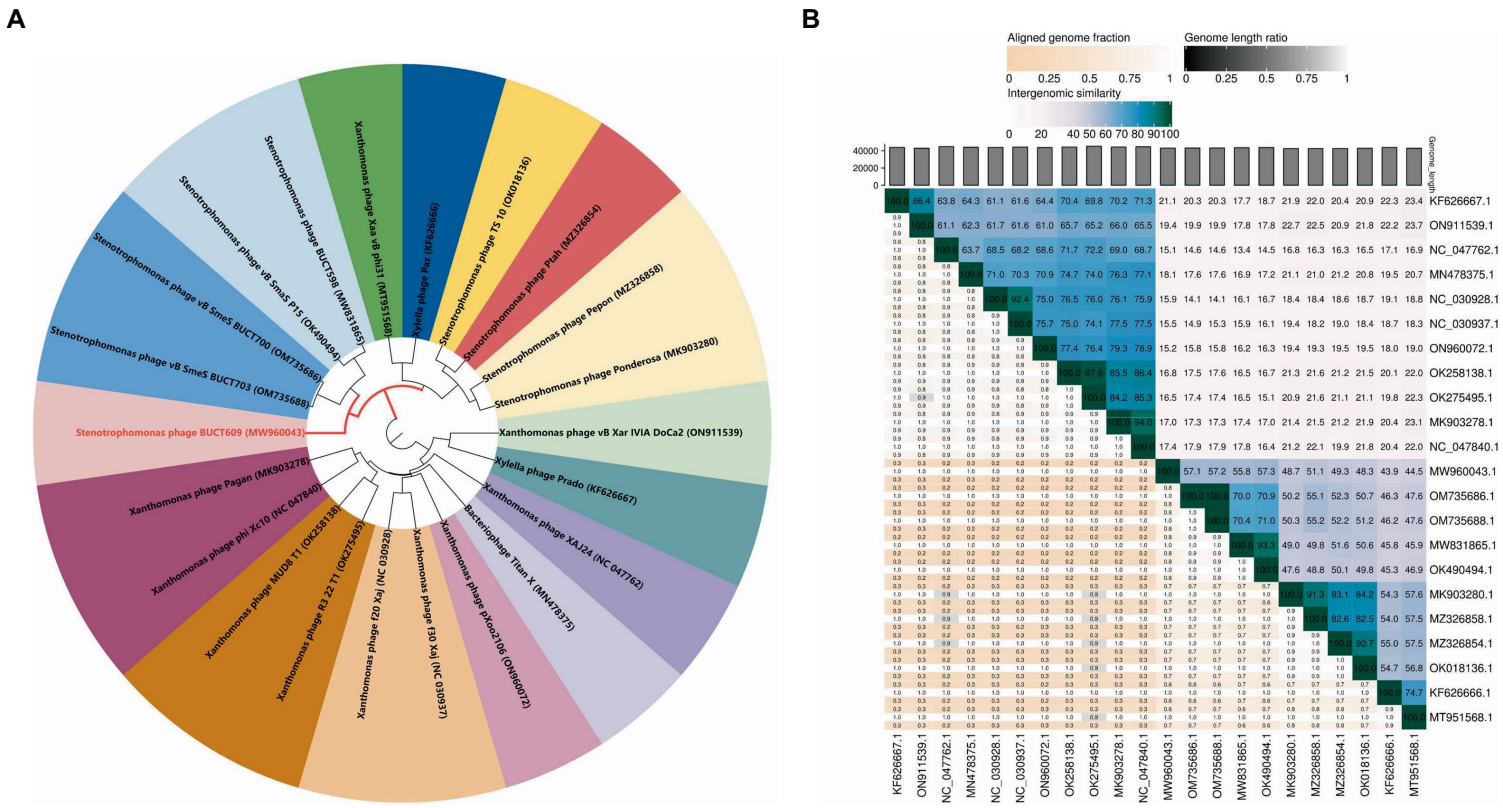
Phylogenetic analysis of phage BUCT609. **(A)** A phylogenetic tree was constructed based on the whole genome sequence by VICTOR which indicated phage BUCT609 could be a new species. **(B)** Percentage sequence similarity between phages calculated using VIDIRIC which shows phage BUCT609 could be a new clade. The horizontal and vertical coordinates indicate the corresponding phage Genebank number.

A total of 21 phages in NCBI had homology (>0%) with BUCT609. To study the evolution of phage BUCT609 and its relationship with other phages, a phylogenetic tree of complete genome sequences of above 22 phages including BUCT609 was constructed. According to VICTOR’s classification of family, genus, and species, 21 strains of phages homologous to phage BUCT609 belonged to the same genus. From NCBI records, all of these strains were presumed to be *Autographiviridae* family and *Pradovirus* genus. However, at the species level, there were 16 different clusters, among which BUCT609 independently represented a species (Figure 4A). To determine whether phage BUCT609 could form an independent clade as a new genus, average nucleotide identity (ANI) analysis with other 21 phages was analyzed. The results showed that BUCT609 had a maximum similarity with *Stenotrophomonas* phage vB_SmaS_P15 (57.3%), which was sufficient for classification at the level of a new genus (Figure 4B).

### Antibacterial effect of BUCT609 *in vivo*

**Figure 5:**
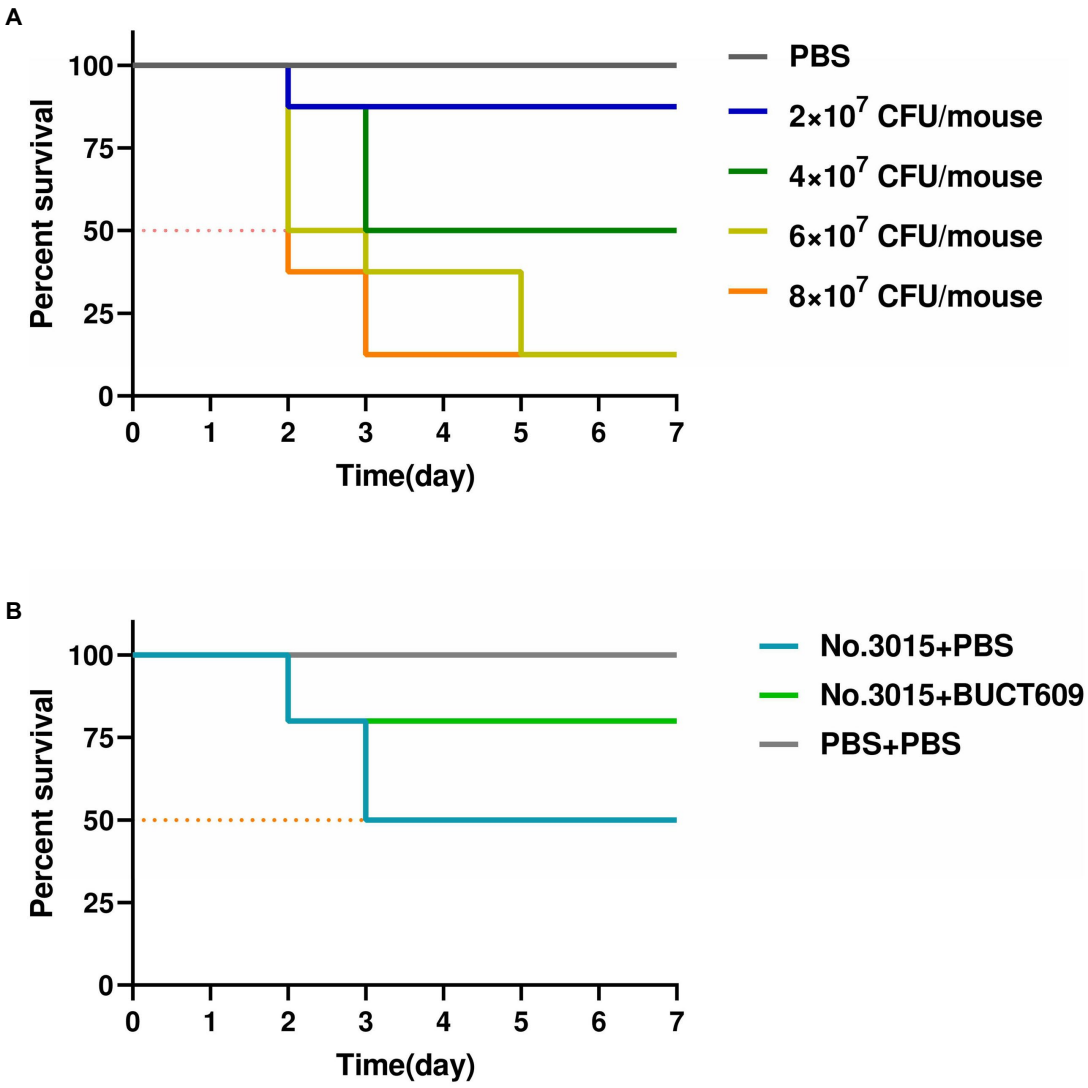
Antibacterial effect of BUCT609 *in vivo*. **(A)** Determination of median lethal dose. The daily percent survival of mice at different titers of bacterial doses. The LD50 was determined as 4 × 10^7^ CFU/ml. When the titer of No.3015 was 4 × 10^7^ CFU/ml, the survival rate of mice was 50%. **(B)** Antibacterial effect of BUCT609 *in vivo*. The daily percent survival of mice before and after treatment. After the treatment, the mice had a survival rate of up to 80%, which was 30% higher than no treatment.

We conducted animal experiments to further explore the possibility of phage BUCT609 for clinical treatment. In mouse models, there was no significant change before and after No.3015 infection (fluctuation within 1 gram). Remarkably, the hair of the mice before bacterial infection was white and shiny while sparse and coarse after infection. Besides, they appeared weaker and clustered together, suggesting that *S. maltophilia* infections could have adverse effects on the organism. The results of infection in mice with different doses were shown in Figure 5A. No mice died in the control group. The survival rate of mice gradually decreased with the increase of injected bacterial titer. When the titer of No.3015 was 4 × 10^7^ CFU/ml, the survival rate of mice was 50%, which was defined as the value of LD50.

The effect of phage BUCT609 in lysing host bacteria *in vivo* was studied by injecting phage BUCT609 to mice infused with LD50. The vital signs of all mice stabilized after 3dpi after the treatment (Figure 5B). After injection of phage BUCT609, the survival rate of mice increased to 80%, which was 30% higher than that of the other group of mice that did not receive treatment. There was no significant change in the weight of the mice before and after phage treatment (fluctuation within 1 gram). Even though the survival rate of the mice improved after the treatment, the coarseness of the hair was not cured. However, the data were sufficient to demonstrate that phage BUCT609 was bacteriostatic *in vivo* and could be used as a potential clinical antimicrobial agent.

## Discussion

Phage BUCT609 was successfully isolated from hospital sewage using *S. maltophilia* No. 3015 as the host bacterium. The host range test showed that 5 of 13 strains of *S. maltophilia* could be lysed by phage BUCT609. Though it does not have a broad host range as phage DLP3, one of the most widespread *S. maltophilia* phages infecting 22 out of 29 *S. maltophilia* strains (Peters et al., 2020), BUCT609 still have a relatively wide host range compared with phage AXL3 that can lyse 5 out of 29 *S. maltophilia* strains (McCutcheon et al., 2020). In previous studies, phage SM1 was found to be effective in animal *S.maltophilia* infection model with 184 PFU/cell burst size (Zhang and Li, 2013). However, the burst size of phage BUCT609 is 382 PFU/cell, which was much higher than that of SM1. Meanwhile, given its temperature stability and wide pH tolerance, BUCT609 shows promising potential to use against multi-drug resistant *S. maltophila* infections. In addition, phylogenetic tree and ANI results showed that phage BUCT609 is an independent new clade that can be classified in subsequent phage discoveries.

For *Autographiviridae* phages that can lyse Gram-negative bacteria, there are two systems for lysing bacteria: the holin-endolysin and pinholin-SAR endolysin pathways (Cahill and Young, 2019). In the former, holin first forms micron-scale pores in the inner membrane, releasing active endolysin into the periplasm to degrade peptidoglycan, thus completing the first step of lysing bacteria. According to genome analysis, the cleavage mechanism of phage BUCT609 was mainly mediated by holin protein encoded by ORF5. Endolysin ORF56 could promote the lysis of host bacteria as well. At present, there are many studies on holin protein, showing that holin protein can be used as a new antibacterial agent. HolGH15, the holin protein in *Staphylococcus aureus* phage GH15, can cause changes in the structural properties of *Listeria monocytogenes* leading to shrinkage, resulting in the release and removal of cellular contents and ultimately leading to the host death. HolGH15 (the final concentration: 240 μg/ml) can reduce *L. monocytogenes* (the initial concentration: 10^6^ CFU/ml) to undetectable levels at 4°C (Song et al., 2021), which shows that the holin protein of phage can potentially become a new type of antibacterial drug by spraying or soaking. BUCT609 can provide a new antibacterial agent in the field of antibacterial as well.

However, phage therapy has many challenges such as safety, ethics, intellectual property rights, and stability (Skurnik et al., 2007). Although this study initially explored the therapeutic effect of phage BUCT609 in mice, the stability of its treatment was not explored. *In vivo* experiments demonstrated that phages can reduce the bacterial load and weight of abscesses (Capparelli et al., 2007). At the same time, increasing evidence suggests that phages may have a major impact on the immune system by interacting with macrophages, neutrophils, and T-cell polarization (Chechushkov et al., 2021). Phage therapy as a potential regimen against drug-resistant bacteria still requires adequate clinical trials.

## Conclusion

Phage therapy has become a new possibility to treat clinically drug-resistant bacteria. The diversity of phages may contribute to the development of phage-based therapies. *In vivo* animal studies demonstrated that phage BUCT609 as a new member to the *S. maltophilia* bacteriophage provided empirical data for phage cocktail therapy in combination with antibiotics against multi-drug resistant bacterial infections.

## Data availability statement

The datasets presented in this study can be found in online repositories. The names of the repository/repositories and accession number(s) can be found in the article/Supplementary material.

## Ethics statement

The animal study was reviewed and approved by the Seventh Medical Center of the PLA General Hospital.

## Author contributions

KH: data curation, writing – original draft, investigation, and validation. YD: data curation, writing – original draf, investigation and validation. LS: supervision and writing – review and editing. ML: supervision, validation, writing – review and editing. HF: conceptualization, methodology, supervision, validation, and writing – review and editing. YT: conceptualization, supervision, validation, and writing – review and editing. All authors contributed to the article and approved the submitted version.

## Funding

This research was supported by Funds for First-class Discipline Construction (nos. XK1805 and XK1803-06), National Key Research and Development Program of China (nos. 2018YFA0903000, 2020YFC2005405, 2020YFA0712100, 2020YFC0840805, 19SWAQ06, 20SWAQX27, and 20SWAQK22), Inner Mongolia Key Research and Development Program (nos. 2019ZD006), NSFC-MFST project (China-Mongolia; no. 31961143024), Fundamental Research Funds for Central Universities (no. BUCTRC201917 and BUCTZY2022).

## Conflict of interest

The authors declare that the research was conducted in the absence of any commercial or financial relationships that could be construed as a potential conflict of interest.

## Publisher’s note

All claims expressed in this article are solely those of the authors and do not necessarily represent those of their affiliated organizations, or those of the publisher, the editors and the reviewers. Any product that may be evaluated in this article, or claim that may be made by its manufacturer, is not guaranteed or endorsed by the publisher.
