# Supplementary material for: Potential application of a newly isolated phage BUCT609 infecting Stenotrophomonas maltophilia
Source: Front Microbiol. 2022 Nov 21;13:1001237. doi: 10.3389/fmicb.2022.1001237 (PMC9720304; doi:10.3389/fmicb.2022.1001237)
Supplement: Supplementary file 1 [file Data_Sheet_1.docx]

| Gene name | Sequence |
| --- | --- |
| atpD | F-ATGAGTCAGGGCAAGATCGTTC |
|  | R-TCCTGCAGGACGCCCATTTC |
| gapA | F-TGGCAATCAAGGTTGGTATCAAC |
|  | R-TTCGCTCTGTGCCTTCACTTC |
| guaA | F-AACGAAGAAAAGCGCTGGTA |
|  | R-ACGGATGGCGGTAGACCAT |
| mutM | F-AACTGCCCGAAGTCGAAAC |
|  | R-GAGGATCTCCTTCACCGCATC |
| nuoD | F-TTCGCAACTACACCATGAAC |
|  | R-CAGCGCGACTCCTTGTACTT |
| ppsA | F-CAAGGCGATCCGCATGGTGTATTC |
|  | R-CCTTCGTAGATGAAGCCGGTGTC |
| recA | F-ATGGACGAGAACAAGAAGCGC |
|  | R-GGTGATGACCTGCTTGAACGG |

**Supplementary Table 1** PCR primer information

**Supplementary Table 2** Phage BUCT609 optimal multiplicity of infection

| Number（No.） | Bacterial concentration (CFU/mL) | Phage concentration (PFU/mL) | Multiplicity of infection (MOI) | 5-h titer (PFU/mL) |
| --- | --- | --- | --- | --- |
| 1 | 10^8^ | 10^10^ | 100 | 3.4×10^8^ |
| 2 | 10^8^ | 10^9^ | 10 | 5.2×10^8^ |
| 3 | 10^8^ | 10^8^ | 1 | 9.1×10^8^ |
| 4 | 10^8^ | 10^7^ | 0.1 | 1.7×10^9^ |
| 5 | 10^8^ | 10^6^ | 0.01 | 3.1×10^9^ |

**Supplementary Table 3** MLST typing results of bacterium and the host range of phage BUCT609

a. (+) and (-) represented that clear and no plaques could be observed after infection with phage, respectively.

| *S. maltophilia* No. | Clear plaques^a^ | ST | Isolation time | Provider |
| --- | --- | --- | --- | --- |
| 118 | + | ST4 | 2012/8/24 | 307 hospital |
| 209 | － | ST463 | 2012/9/25 | 307 hospital |
| 532 | － | ST296 | 2013/5/14 | 307 hospital |
| 548 | + | ST190 | 2013/5/22 | 307 hospital |
| 690 | － | ST115 | 2013/8/9 | 307 hospital |
| 824 | － | ST413 | 2013/10/23 | 307 hospital |
| 826 | － | ST378 | 2013/10/24 | 307 hospital |
| 992 | + | ST8 | 2014/3/13 | 307 hospital |
| 1207 | + | ST502 | 2014/7/19 | 307 hospital |
| 1209 | － | ST7 | 2014/4/25 | 210 hospital |
| 1785 | － | ST31 | 2015/11/11 | 210 hospital |
| 1786 | － | ST362 | 2015/11/11 | 210 hospital |
| 3015 | + | ST95 | 2021/4/13 | Shanghai Public Health Clinical Center |

**Supplementary Table 4** Sensibility of *S. maltophilia* strain No.3015 to 12 kinds of antibiotics

| Antibiotics | Zone of inhibition (mm) | Sensibility |
| --- | --- | --- |
| cefalexin | - | R |
| erythromycin | - | R |
| roxithromycin | - | R |
| ceftriaxone | 11 | S |
| cefazolin | - | R |
| cephalothin | - | R |
| tetracycline | 28 | S |
| cefaclor | - | R |
| Norfloxacin | - | R |
| Trimethoprim | 27 | S |
| Minocycline | 26 | S |
| Levofloxacin | 26 | S |

Symbols: (−) no zone of inhibition after infection.
